# Derivation and validation of a clinical model to identify cryptococcosis from suspected malignant pulmonary nodules: A dual‐center case‐control study

**DOI:** 10.1002/ctm2.544

**Published:** 2021-10-12

**Authors:** Bei Mao, Hai Zhang, Wen‐Wen Wang, Hai‐Wen Lu, Jia‐Wei Yang, Sen Jiang, Xiao‐Dan Ye, Feng Li, Jin‐Fu Xu

**Affiliations:** ^1^ Department of Respiratory and Critical Care Medicine Shanghai Pulmonary Hospital Institute of Respiratory Medicine School of Medicine Tongji University Shanghai China; ^2^ Department of Pulmonary Medicine Shanghai Chest Hospital Shanghai Jiao Tong University Shanghai China; ^3^ Department of Radiology Shanghai Pulmonary Hospital Tongji University School of Medicine Shanghai China; ^4^ Department of Radiology Shanghai Chest Hospital Shanghai Jiao Tong University Shanghai China

Dear Editor,

Pulmonary cryptococcosis (PC) usually presents as single or multiple pulmonary nodules (PNs) in immunocompetent patients, easily mimicked lung cancer clinically and radiologically (Figure 1), caused many unnecessary surgeries. To settle this problem, we established and validated a clinical model to identify PC from suspected malignant PNs.

**FIGURE 1:**
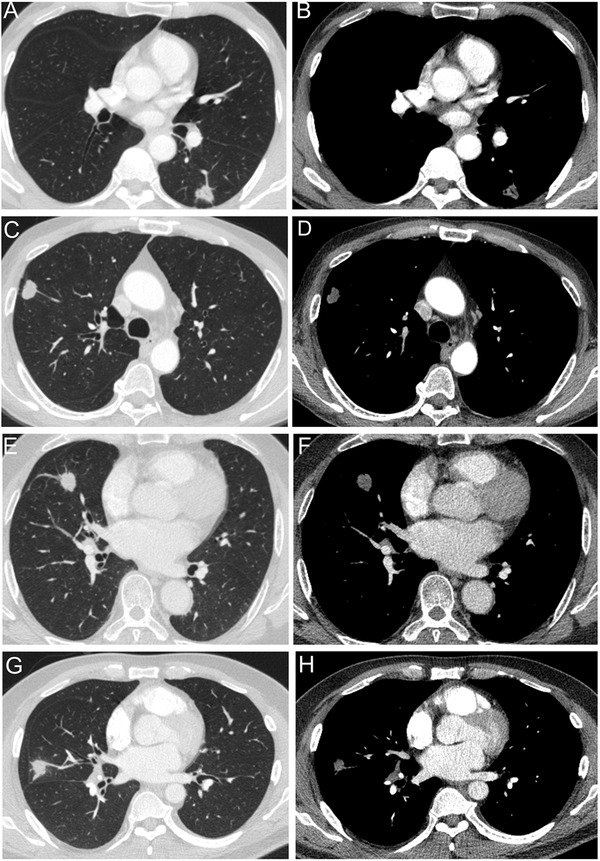
Examples of representative CT images. (A, B) Male, 65 years old, suspected malignant nodule, and pathological diagnosed as pulmonary cryptococcosis. (C, D) Male, 61 years old, long history of smoking, suspected malignant nodule and pathological diagnosed as pulmonary cryptococcosis. (E, F) Male, 57 years old, suspected pulmonary cryptococcosis, and pathological diagnosed as pulmonary cryptococcosis adenocarcinoma. (G, H) Male, 52 years old, suspected pulmonary cryptococcosis, and pathological diagnosed as pulmonary cryptococcosis adenocarcinoma

PC is caused by *Cryptococcus* spp., a fungal disease that is endemic in many countries.^1^ Previously though, PC occurred in immunocompromised patients, such as infected by human immunodeficiency virus, long‐term use of immunosuppressant, it's also common in immunocompetent population. PC was easily misdiagnosed as malignancy in immunocompetent patients, due to the relatively lower positive rate of culture or antigen detection of *Cryptococcus* in the patients with localized lesion.^2^, ^3^ Previous studies showed that over 30% of PC were misdiagnosed as malignancy and suffered unwanted surgery.^4^, ^5^

In the present study, we performed a multicenter case‐control study and two specialized pulmonary hospitals with a large amount of lung surgery, Shanghai Pulmonary Hospital and Shanghai Chest Hospital participated in this study. Patients with suspected malignant PNs, subsequent surgical histopathological diagnosed as PC from centers from January 2014 to March 2019 were included. Patients with suspected malignant PNs and surgical histopathological diagnosed as malignancy from centers between December 2018 and March 2019 were also included. A standardized data collection spreadsheet was designed to obtain patient's general and anthropometric information, comorbidities, laboratorial indicators from electronic medical records. Radiological images when the PNs was first detected were obtained from medical system and reevaluated by two radiologists with more than 20 years of experience without knowing the final diagnosis. Ethics Committee of Shanghai Pulmonary Hospital (K20‐006) and Shanghai Chest Hospital (IS2006) approved this study.

Total 1042 suspected malignant PNs were finally included and then randomly split into derivation (364 PC and 383 malignancy) and validation set (142 PC and 153 malignancy) at a ratio of 7:3. Based on derivation set, the following predictors of PC were identified according to the logistic regression: male (OR: 1.95, 95%CI: 1.08‐3.52), located in lower lobe (OR: 2.72, 95%CI: 1.50‐4.92), morphological irregularity (OR: 7.06, 95%CI: 3.73‐13.36), the presence of Halo sign (OR: 33.62, 95%CI: 15.58‐72.56) and Feeding vessel sign (OR: 2.34, 95%CI: 1.26‐4.32), part‐solid (OR: 5.99, 95%CI: 1.82‐19.76) or solid type (OR: 41.73, 95%CI: 12.68‐137.32). The screening process of predictive indicators is shown in Supplementary tables. RAS score (Radiography, Age, and Sex) was established according to β regression coefficients estimated from the logistic model (Table 1). The RAS score ranges from ‐6 to 13 points and patients with higher scores have a higher probability of PC. After validated, RAS score showed good discrimination with an AUC of 0.982 (95%CI: 0.971‐0.993) (Figure 2A). Furthermore, visual inspection of the calibration plot and curve showed good agreement between RAS score predicted probabilities of PC and actual prevalence of PC in PNs (Figures 2B and 2C).

**TABLE 1:** Final score and scoring of each variable in the score system

Predictive marker	RAS points
Age, years	
<40	0
40–59	–1
≥60	–2
Sex	
Female	0
Male	1
Location	
Other lobes	0
Lower lobe	1
Morphology	
Round or roundish	0
Irregularity	2
Density	
pGGN	0
Part‐solid	2
Solid	4
Spiculation	
No	0
Yes	–3
Halo sign	
No	0
Yes	4
Vacuole sign	
No	0
Yes	–1
Feeding vessel sign	
No	0
Yes	1

pGGN: pure ground glass nodule.

**FIGURE 2:**
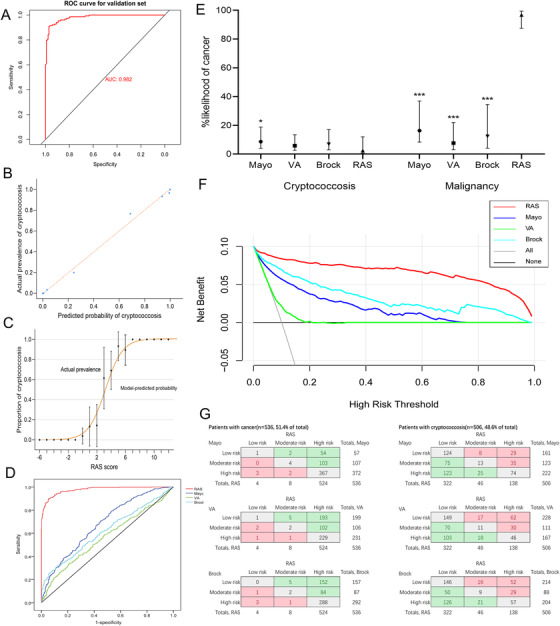
Validation of RAS model and performance comparison of these models. (A) Discrimination of RAS model. (B) Calibration plots of RAS model. (C) Calibration curves by plotting the predicted probabilities of cryptococcosis divided into 20 groups based on the RAS score. Data are presented as mean and 95%CI. (D) Comparison of discrimination in these models. (E) Comparison of prediction accuracy in these models. Data were presented as median with IQR. (F) Analysis of clinical net benefit based on these models. (G) Net reclassification improvement of RAS model compared with previous clinical models

Three models (Mayo Clinic,^6^ Veterans Association [VA],^7^ and Brock University^8^) were widely quoted to estimate the probability of malignant nodules in clinical practices and guidelines. However, the ability of these models to distinguish PC and malignancy remains unclear. We further compared the performance of these models with RAS model in the entire dataset in this study. The results showed that these models both underperformed the RAS model on the accuracy of malignancy prediction and the discriminative ability of malignancy and PC (Figures 2D and 2E). Moreover, decision curve analysis was used to analyze the clinical net benefit based on these models. Results showed that RAS score provided a larger net benefit than Mayo, VA and Brock models (Figure 2F). Furthermore, net reclassification improvement (NRI) was used to analyze the improvement in classification of cryptococcosis and malignancy in these models based on the following risk classification: <5% is low‐risk, ≥10% is high‐risk, and moderate‐risk is ≥5% to <10%. After calculation, the additive and absolute NRIs of RAS model were 58.6% and 29.3% compared with Mayo model, 71.4% and 36.3% compared with VA model, 63.8% and 32.3% compared with Brock model (Figure 2G). This result suggested that RAS model displayed improved risk stratification than other models.

In this study, the stronger predictors of cryptococcosis in patients with suspected malignant PNs were the presence of Halo sign and solid density in chest CT images. Halo sign is described as ground‐glass opacity around the nodules and may be caused by lung inflammatory infiltration. Halo sign is nonspecific to cryptococcosis and represents granulomatous inflammation on histological examination.^9^, ^10^ Another solid density dominated the predictive factor of cryptococcosis indicated that the HRCT plays an important role in distinguish malignancy from benign nodules, especially cryptococcosis, which is resembling a malignancy. What is noteworthy is that nodule size was not a predictor for differentiating malignancy and cryptococcosis in this study, mainly because the nodule size of cryptococcosis often ranges from micronodules to mass. However, nodule size is one of the influence factors in medical choice such as percutaneous lung biopsy or surgical resection in the clinical practice. Physicians should comprehensively consider all relevant indicators, rather than only focus on the nodule size, before deciding on invasive interventions, which could provide better individualization project for those patients.

Although previous models have been tested in some independent cohorts to help predicting the probability of malignant nodules, these models have not been evaluated in distinguishing cryptococcosis and malignant nodules. The most important reason for different performance between these models is the indefinite final diagnosis of many nodules in these studies. Theoretically, models that were developed based on definite diagnosis have high accuracy and authenticity. Although these models performed well in differentiating benign and malignant nodules, they performed poorly in differentiating cryptococcosis and malignant nodules, indirectly demonstrating that PC easily mimicked and misdiagnosed malignant nodules.

In summary, we established and validated a clinical model that accurately identifies cryptococcosis from suspected malignant PNs. While comprehensively assessing patient's living environment and the dynamic course of the disease, the RAS score can be applied to identify cryptococcosis from suspected malignant nodules, which may be helpful to avoid unwanted surgery and guide decision making.

## CONFLICT OF INTEREST

The authors declare that there is no conflict of interest.

## Supporting information

SUPPORTING INFORMATION

SUPPORTING INFORMATION

SUPPORTING INFORMATION
