# Supplementary material for: Derivation and validation of a clinical model to identify cryptococcosis from suspected malignant pulmonary nodules: A dual‐center case‐control study
Source: Clin Transl Med. 2021 Oct 12;11(10):e544. doi: 10.1002/ctm2.544 (PMC8506637; doi:10.1002/ctm2.544)
Supplement: Supplementary file 1 — SUPPORTING INFORMATION [file CTM2-11-e544-s003.docx]

Supplementary Table 1. Demographics, comorbidities and laboratorial characteristics of pulmonary cryptococcosis and lung cancer in derivation set.

|  | **Pulmonary cryptococcosis** | **Lung cancer** | **P** |
| --- | --- | --- | --- |
| N | 364 | 383 |  |
| Demographics |  |  |  |
| Male, % | 218 (59.9%) | 139 (36.3%) | **<0**·**001***** |
| Age, years | 52 (15) | 59 (16) | **<0**·**001***** |
| BMI, kg/m^2^ | 24·4 (SD 3·0) | 23·7 (SD 3·3) | 0·092 |
| Current smoker | 54 (14·8%) | 47 (12·3%) | 0·306 |
| Comorbidities |  |  |  |
| Hypertension | 74 (20·3%) | 85 (22·2%) | 0·534 |
| Diabetes mellitus | 35 (9·6%) | 38 (9·9%) | 0·888 |
| Autoimmune disease | 7 (1·9%) | 7 (1·8%) | 0·923 |
| Extrapulmonary malignancy | 16 (4·4%) | 16 (4·2%) | 0·883 |
| Organ transplantation | 0 | 0 | - |
| Long-term use of glucocorticoid or immunosuppressor | 0 | 0 | - |
| Laboratorial |  |  |  |
| Hemoglobin, g/L | 139 (21) | 136 (19) | 0·021* |
| WBC, 10^9/L | 5·8 (2·3) | 5·9 (1·9) | 0·775 |
| Neutrophils% | 59·6 (10·1) | 60·9 (16·1) | 0·091 |
| Eosinophils% | 1·8 (1·9) | 1·4 (1·7) | **0**·**017*** |
| CEA, μg/L | 1·3 (1·1) | 1·3 (1·3) | 0·423 |
| NSE, ng/ml | 12·6 (2·9) | 12·1 (2·9) | 0·068 |
| CA153, U/ml | 8·9 (8·3) | 8·3 (5·0) | 0·084 |
| CYFRA21-1, ng/ml | 1·3 (0·8) | 1·1 (0·8) | 0·102 |
| SCCAg, ng/ml | 1·1 (0·1) | 1·1 (0·2) | 0·106 |
| Albumin, g/L | 42 (5) | 43 (5) | 0·196 |
| D-dimer, ng/ml | 109 (113·3) | 111 (79·5) | 0·271 |

Data are n (%), mean (SD), or median (IQR). BMI: Body Mass Index; WBC: White Blood Cells; CEA: Carcinoma Embryonic Antigen; NSE: Neuron Specific Enolase; SCCAg: Squmaous Cell Carcinoma Antigen.
