# Supplementary material for: Derivation and validation of a clinical model to identify cryptococcosis from suspected malignant pulmonary nodules: A dual‐center case‐control study
Source: Clin Transl Med. 2021 Oct 12;11(10):e544. doi: 10.1002/ctm2.544 (PMC8506637; doi:10.1002/ctm2.544)
Supplement: Supplementary file 2 — SUPPORTING INFORMATION [file CTM2-11-e544-s001.docx]

Supplementary Table 2. Radiologic characteristics of pulmonary cryptococcosis and lung cancer in derivation set.

|  | **Pulmonary cryptococcosis** | **Lung cancer** | **P** |
| --- | --- | --- | --- |
| N | 364 | 383 |  |
| Size, mm | 12·0 (7·0) | 10·0 (9·0) | 0·059 |
| Site |  |  | **<0**·**001***** |
| Left upper lobe | 57 (15·7%) | 109 (28·5%) |  |
| Left lower lobe | 89 (24·5%) | 69 (18·0%) |  |
| Right upper lobe | 77 (21·2%) | 129 (33·7%) |  |
| Right middle lobe | 19 (5·2%) | 27 (7·0%) |  |
| Right lower lobe | 122 (33·5%) | 49 (12·8%) |  |
| Subpleural | 211 (58·0%) | 204 (53·3%) | 0·196 |
| Morphology |  |  | **<0**·**001***** |
| Round or roundish | 133 (36·5%) | 305 (79·6%) |  |
| Irregularity | 231 (63·5%) | 78 (20·4%) |  |
| Edge |  |  |  |
| Unclear | 247 (67·9%) | 115 (30·0%) | **<0**·**001***** |
| Lobulated | 155 (42·6%) | 141 (36·8%) | 0·107 |
| Density |  |  | **<0**·**001***** |
| Solid | 257 (70·6%) | 80 (20·9%) |  |
| part-solid | 98 (26·9%) | 177 (46·2%) |  |
| pGGN | 9 (2·5%) | 126 (32·9%) |  |
| Sign |  |  |  |
| Halo sign | 273 (75%) | 38 (9·9%) | **<0**·**001***** |
| Spiculation | 151 (41·5%) | 336 (87·7%) | **<0**·**001***** |
| Clustered | 43 (11·8%) | 35 (9·1%) | 0·232 |
| Vacuole sign | 46 (12·6%) | 75 (19·6%) | **0**·**01**** |
| Feeding vessel sign | 190 (52·2%) | 131 (34·2%) | **<0**·**001***** |
| Pleural pull | 122 (33·5%) | 134 (35·0%) | 0·672 |
| Lymphadenectasis | 21 (5·8%) | 14 (3·7%) | 0·172 |

Data are n (%), mean (SD), or median (IQR). pGGN: pure ground glass nodule.
