# Supplementary material for: Derivation and validation of a clinical model to identify cryptococcosis from suspected malignant pulmonary nodules: A dual‐center case‐control study
Source: Clin Transl Med. 2021 Oct 12;11(10):e544. doi: 10.1002/ctm2.544 (PMC8506637; doi:10.1002/ctm2.544)
Supplement: Supplementary file 3 — SUPPORTING INFORMATION [file CTM2-11-e544-s002.docx]

Supplementary Table 3. Screening of predictive indicators for cryptococcosis in logistic regression.

| Predictive Marker | Unadjusted | | Fully Adjusted | |
| --- | --- | --- | --- | --- |
|  | OR (95%CI) | P | OR (95%CI) | P |
| Age, yrs | 0·96 (0·95, 0·97) | <0·001*** | 0·95 (0·92, 0·97) | <0·001*** |
| Sex |  |  |  |  |
| Female | Ref | Ref | Ref | Ref |
| Male | 2·62 (1·95, 3·52) | <0·001*** | 1·95 (1·08, 3·52) | 0·026* |
| Size, mm | 1·00 (0·98, 1·03) | 0·658 |  |  |
| Lower lobe |  |  |  |  |
| No | Ref | Ref | Ref | Ref |
| Yes | 3·10 (2·29, 4·18) | <0·001*** | 2·72 (1·50, 4·92) | 0·001*** |
| Morphological irregularity |  |  |  |  |
| No | Ref | Ref | Ref | Ref |
| Yes | 6·79 (4·89, 9·43) | <0·001*** | 7·06 (3·73, 13·36) | <0·001*** |
| Edge clear |  |  |  |  |
| No | Ref | Ref | Ref | Ref |
| Yes | 0·20 (0·15, 0·23) | <0·001*** | 0·73 (0·37, 1·46) | 0·376 |
| Halo sign |  |  |  |  |
| No | Ref | Ref | Ref | Ref |
| Yes | 27·24 (18·07, 41·06) | <0·001*** | 33·62 (15·58, 72·56) | <0·001*** |
| Spiculation |  |  |  |  |
| No | Ref | Ref | Ref | Ref |
| Yes | 0·10 (0·07, 0·14) | <0·001*** | 0·08 (0·04, 0·15) | <0·001*** |
| Vacuole sign |  |  |  |  |
| No | Ref | Ref | Ref | Ref |
| Yes | 0·59 (0·40, 0·89) | 0·011* | 0·38 (0·17, 0·83) | 0·015* |
| Feeding vessel sign |  |  |  |  |
| No | Ref | Ref | Ref | Ref |
| Yes | 2·10 (1·56, 2·82) | <0·001*** | 2·34 (1·26, 4·32) | 0·007** |
| Density |  |  |  |  |
| pGGN | Ref | Ref | Ref | Ref |
| Part-solid | 7·75 (3·77, 15·92) | <0·001*** | 5·99 (1·82, 19·76) | 0·003** |
| Solid | 44·98 (21·86, 92·52) | <0·001*** | 41·73 (12·68, 137·32) | <0·001*** |

Ref: reference; pGGN: pure ground glass nodule.
